# Preparation and in Vitro Evaluation of New Composite Mesh Functionalized with Cationic Antimicrobial Peptide

**DOI:** 10.3390/ma12101676

**Published:** 2019-05-23

**Authors:** Pengbi Liu, Nanliang Chen, Jinhua Jiang, Xuejun Wen

**Affiliations:** 1 College of Textiles, Donghua University, Shanghai 201620, China; 1135009@mail.dhu.edu.cn; 2 Department of Chemical and Life Science Engineering, School of Engineering, Virginia Commonwealth University, Richmond, VA 23284, USA; 3 School of Pharmaceutical Sciences, Guangzhou Medical University, Guangzhou 511436, China

**Keywords:** antimicrobial peptide, surgical mesh, antibacterial, cytotoxicity, electrospinning, hernia repair, in vitro release, mechanical properties

## Abstract

Infection caused by bacteria in hernia repair site is a severe complication, and patients have to undergo a second surgery to remove the infected prosthesis. In this study, we developed a composite biological safe mesh with antibacterial activity. The composite mesh is composed of large pore polypropylene (PP) mesh, poly-caprolactone (PCL) and antimicrobial peptide (PEP-1), which we synthesized in our lab. Fourier transformed infrared (FTIR) spectroscopy was utilized to analyze the functional groups. The surface morphology, in vitro release characters, mechanical properties, antibacterial activities, and in vitro cytotoxicity of modified mesh were evaluated. Results showed that PEP-1 was loaded in fibers successfully and could diffuse from nanofibers to inhibit bacteria (*E. coli*) growth. However, the modified mesh did not show inhibition to *S. aureus*. The mechanical properties of fabricated mesh showed no difference with two commercial surgical meshes. What is more, modified mesh was proved to be nontoxic to human dermal fibroblasts, indicating that this method to fabricate meshes with antibacterial activity is feasible and provides a new strategy for the development of surgical meshes.

## 1. Introduction

Polypropylene (PP) meshes have been widely used in hernia repair surgical fields since their appearance in the 1950s [1]. Many researches have reported that the utilization of surgical meshes decreases hernia recurrences [2,3]. However, some potential mesh-related complications like chronic pain, mechanical failure and infections are also nonnegligible [4,5,6,7]. Robinson et al. reported that the infection complications in all mesh types reached 42% (107 reports in 252 cases) [6]. While Arnold et al. found that only three surgical site infections occurred in 1424 laparoscopic inguinal hernia repairs performed with a three-dimensional contoured mesh [8]. The infection rate varied by hernia site; for instance, the incidence of infection in ventral hernia was about 8.3% [9] and that of groin was about 0.3% [10]; however, the crude infection rate was about 5% [7]. Though the infection rate is not very high, in most cases the infected meshes have to be removed; this requires a secondary surgery and causes a lot of pain and costs to patients [11]. Therefore, meshes with appropriate mechanical properties and anti-infection functions are urgently needed.

It has been found that the most common bacteria that appear in infections of hernia sites are *S. aureus* (SA), *S. epidermidis*, *E. coli* (EC), *Pseudomonas aeruginosa* and *Streptococcus pyogenes* [12,13,14,15]. Therefore, many studies have attempted to fabricate meshes with antimicrobial properties. Labay et al. conferred PP meshes with antibacterial activity by loading ampicillin to the surface of plasma-treated PP monofilaments [16]. What is more, Guillaume and co-workers modified PP meshes with multiple layers of biodegradable polymers containing ofloxacin and rifampicin with an airbrush spray system [17]. These methods of loading antimicrobial agents to meshes are successful to inhibit the growth of many bacterial strains. However, with the overuse of antibiotics, many multidrug-resistant and persistent bacteria appeared, which complicates medical treatment and threatens public health [18,19,20].

Antimicrobial peptides (AMPs), also known as host defense peptides, have attracted more and more attentions from scientists over the world in recent years and are deemed to be a new promising generation of antimicrobial agents [21,22,23,24,25,26]. Over 3000 AMPs have been documented in the AMP database so far, including natural and synthetic peptides [27]. While unlike traditional antibiotics, which mainly target some specific intracellular sites of pathogens, most small cationic antimicrobial peptides generally target bacterial cell membranes initially via electrostatic interaction and then destroy membranes or interfere with the synthesis of enzymes, proteins, cell wall or nucleic acids to inhibit microorganisms [28,29,30,31,32,33,34]. Therefore, many AMPs exhibit broad-spectrum bactericidal activities against Gram-positive bacteria, Gram-negative bacteria and fungi, including some multidrug-resistant bacteria [35,36]. Considering these advantages, AMPs may also be used in hernia repair surgical meshes.

In this work, we investigated the antibacterial efficiency and cytotoxicity of antimicrobial peptide (PEP-1)-loaded composite mesh. Poly-caprolactone(PCL) was selected as a media to mix with PEP-1 due to its brilliant biocompatibility and ability to degrade in the body. We hypothesized that peptides would diffuse from electrospinning PCL nanofibers sustainably and therefore confer nanosheets with antibacterial properties. The in vitro peptide release profiles, mechanical properties, antibacterial activities, and in vitro cytotoxicity of mesh were evaluated.

## 2. Materials and Methods

### 2.1. Materials

Hexafluoro-2-propanol (HFIP) was purchased from Oakwood, West Columbia, SC, USA. Poly(Ɛ-caprolactone) (PCL, Mn = 80,000), glutaraldehyde, ethanol, dimethyl sulfoxide (DMSO) and phosphate buffered saline (PBS) tablets were supplied by Sigma-Aldrich (St. Louis, MO, USA). Hank’s balanced salt solution (HBSS) was obtained from Thermo Fisher Scientific, USA. LB-broth and LB-Agar (Fisher Scientific, USA) were used to prepare the bacterial culture media. Antimicrobial peptide PEP-1 (RRRGRRRGPPGRRRGRRR) was synthesized via standard Fmoc-solid phase peptide synthesis protocol and purified in our lab with a purity of 98% as determined by HPLC. The peptide synthesis and reverse-phase high performance liquid chromatography (RP-HPLC) analysis and purification process are described in the Supplementary Material. The matrix material used in this study was the large pore polypropylene mesh R we investigated before [37].

Human dermal fibroblasts (HDFs) were provided by ScienCell Research Laboratories, Inc., USA. Cell culture medium was Dulbecco’s modified Eagle’s medium (DMEM/F12+GlutaMAX^TM^-1, Gibco BRL Life Technologies, USA) containing 10% fetal bovine serum and 1% Penicillin/Steptomycin (both from Sigma Aldrich, St. Louis, MO, USA).

The microorganisms used here were standard strains *S. aureus* (SA, ATCC 6538, Gram positive) and *E. coli* (EC, ATCC 25922, Gram negative). These two bacteria species were chosen because they are the main bacteria found at the tissue infection sites [34,38].

LIVE/DEAD™ Reduced Biohazard Cell Viability Kit was purchased from Invitrogen^TM^ (Carlsbad, CA, USA). Alamar Blue^TM^ reagent was obtained from VWR, USA.

### 2.2. Preparation of Composite Mesh

Composite scaffolds prepared in this work are referred to as AMP-PCL. Ten percent (*w*/*v*) PCL in HFIP was firstly prepared for further study. Sixty milligrams of PEP-1 was dissolved into 1 mL 10% PCL solution and stirred overnight, and then the mixture was electrospun onto the PP mesh for 30 min to obtain AMP-PCL. During the electrospinning, solutions were fed at a rate of 25 µL/min. The high voltage applied to the 23 G blunt needle was 13 kV, and the drop height was 10 cm. Fabricated sheets were vacuumed for 7 days to eliminate the influence of organic solvents.

### 2.3. Field-Emission Scanning Electron Microscopy

SEM images were obtained on a Hitachi SU-70 Field-Emission Scanning Electron Microscope (SEM, Hitachi SU-70, Tokyo, Japan). Before imaging, the scaffolds were mounted on SEM sample holders and sputter-coated with a thin layer of gold and palladium mixture. Images were analyzed by Nano Measurer 1.2 (Fudan University, Shanghai, China).

### 2.4. FTIR Spectroscopy

To determine the existence of peptides in scaffolds, a fourier transformed infrared (FTIR) spectroscopy (NICOLET iS10, Thermo scientific, Waltham, MA, USA) was utilized. The frequency region was set as 500–4000 cm^−1^.

### 2.5. Tensile Strength Test

To evaluate the mechanical properties, samples were cut into 10 × 45 mm pieces along the transverse (weft) or longitudinal (warp) directions and gripped to a tensile tester (Shimadzu EZ Graph, Nakagyoku, Kyoto, Japan) with an initial gauge length of 15 mm. Samples were tested at a speed of 25 mm/min and the elastic modulus and max stress data were collected and recorded as mean ± standard deviation. The matrix PP mesh and two commercial surgical meshes (PROLENE^TM^ Soft and PROCEED^TM^ Surgical mesh, Ethicon, Inc, Somerville, NJ) were also tested for comparisons. Each test was repeated five times. Differences between mesh types were evaluated using a one-way analysis of variance (ANOVA) with Tukey’s post-test. Comparisons with *p* < 0.05 were considered statistically significant.

### 2.6. In Vitro Release

Release of PEP-1 from scaffolds in vitro was evaluated following the previous methods [39,40]. Firstly, the standard curve of peptides was produced by measuring the absorption value of different concentrations at 214 nm by NanoDrop™ 2000 Spectrophotometers (Thermo Scientific™, Waltham, MA, USA). Then, 12 mg AMP-containing PCL nanofiber film, containing about 4.5 mg PEP-1, was peeled off from the mesh and immersed into 1 mL PBS buffer and incubated at 37 °C with shaking. Subsequently, 100 μL of the supernatant was taken at predetermined time intervals from the release medium and replaced with the same volume of PBS buffer. The collections were measured by NanoDrop™ 2000 Spectrophotometers at 214 nm to analyze the released peptides.

### 2.7. Antibacterial Activity

Before the antibacterial test, AMP-PCL was sterilized by immersing into 75% ethanol for 30 min. The antibacterial efficacy of the PEP-1-loaded scaffolds was tested against EC and SA bacteria. In the current study, an agar diffusion method was adapted from the references with a little modification [39,41,42,43]. Firstly, SA and EC bacteria were cultured two passages in LB-broth after cell thawing from frozen storage. Then bacteria density was diluted to 1~5 × 10^6^ CFU/mL, and 100 µL bacteria solution was spread evenly onto a LB-Agar plate. Samples were cut into 10 × 10 mm pieces, containing approximately 0.45 mg PEP-1, under sterile conditions and put on the LB-Agar plates carefully (n = 4). After being incubated at 37 °C for 48 h, the distance between the mesh edge and inhibition zone edge was measured. PP mesh with PCL electrospun film was used as the control and sterilized with the same method.

### 2.8. Cytotoxicity Assay

To investigate the in vitro cytotoxicity, the cell viability of HDFs cultured in the leaching liquid of meshes was evaluated based on the International Standards (ISO 10993-5) [44]. Briefly, 10 × 10 mm pieces of samples (n = 5) were prepared and immersed in 2 mL of cell culture medium in a 6-well cell culture plate (Cellstar^®^, Greiner Bio-One) for 24 h at 37 °C to get extracts. HDFs were collected and seeded in a 48-well cell culture plate. 1.5 × 10^4^ cells and 200 µL cell culture medium were placed into each well and cultured for 24 h, then the medium was replaced with equivalent leaching liquid and incubated for another 24 h before further study. Ten percent of DMSO in cell culture medium was set as the positive control. And the complete cell culture medium was set as another control.

Cell viability was measured by Alamar Blue assay. After 24 h of incubation, 20 µL of Alamar Blue was added to each well and incubated for 4 h before fluorescence reading (Synergy H1 Hybrid reader, BioTek, USA). The excitation wavelength and emission wavelength were set at 540 nm and 570 nm, respectively. Fifty microliters of Alamar Blue in 500 µL of cell culture medium without cells was used as blank control.

Finally, cell morphology was observed by a confocal laser scanning microscope (Olympus IX81, Tokyo, Japan). Cells were washed with HBSS after Alamar Blue assay, then treated with a mixture of Live/Dead reagents for 15 min at room temperature. Four percent of glutaraldehyde in HBSS was freshly prepared to fix cells for 1 h before fluorescence imaging.

## 3. Results

### 3.1. Composite Mesh Surface Morphology

**Figure 1:**
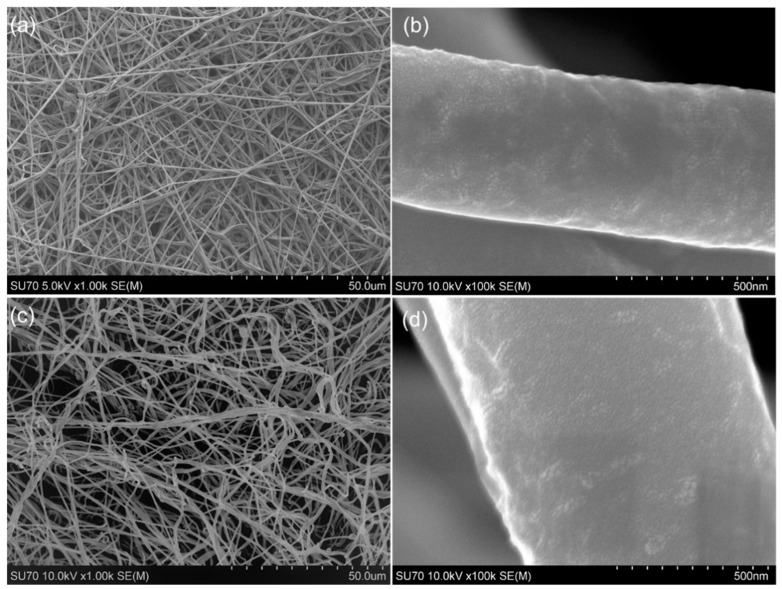
Surface morphology of scaffolds by SEM. (**a**) PCL nanofiber film, ×1.00 k; (**b**) PCL nanofiber film, ×100 k; (**c**) AMP-PCL surface, ×1.00 k; (**d**)AMP-PCL surface, ×100 k.

As shown in Figure 1, the average diameter of PCL nanofibers was 569.83 ± 56.89 nm, and 552.94 ± 76.14 nm for that of AMP-contained nanofibers. Diameters of PCL and AMP-PCL fibers showed no significant difference with each other, and the magnified graphs suggest that their surface conditions are also similar (Figure 1b,d).

### 3.2. Fourier Transform Infrared Spectroscopy

**Figure 2:**
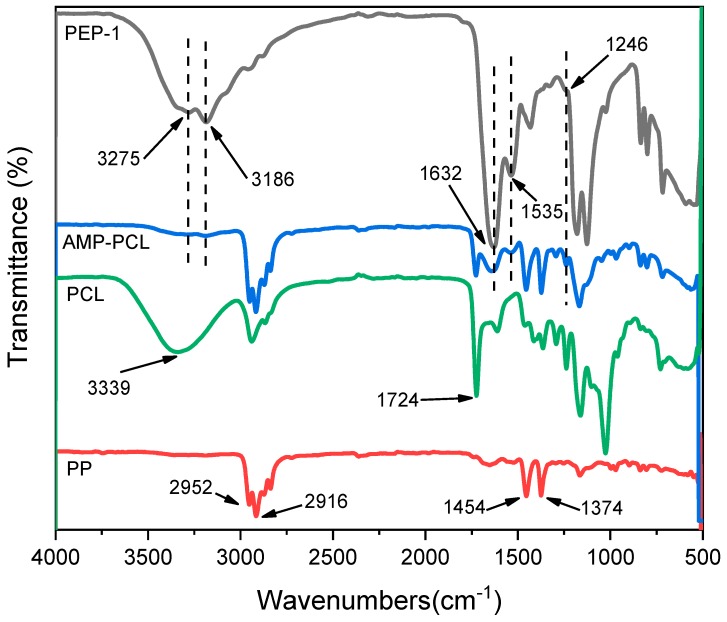
Fourier transform infrared spectra of fabricated scaffolds and original samples.

The FTIR spectra of AMP-PCL and the original materials are displayed in Figure 2. The peaks appearing on the PP spectrum are 2916 cm^−1^ (v*_a_* CH_2_), 2952 cm^−1^ (v*_a_* CH_3_), 1374 cm^−1^ (δCH_3_) and 1454 cm^−1^ (δCH_3_) [45]. The peaks appearing at 1724 cm^−1^ are the stretching of the ester carbonyl, the typical peak of PCL [46]. The broad peaks at 3339 cm^−1^ on PCL spectrum are due to the presence of the hydroxyl groups [46]. In the case of PEP-1, absorbance bands at 1632 cm^−1^, 1535 cm^−1^ and 1246 cm^−1^ confirmed amide I (C=O stretching), amide II (CN stretching, NH bending), and amide III (CN stretching, NH bending), respectively [47]. Peaks at 3186 cm^−1^ and 3275 cm^−1^ are the NH stretching vibration (ν NH) of amide groups. All the amide absorbance bands appeared on the spectrum of AMP-PCL scaffolds, suggesting that peptides were loaded in scaffolds successfully.

### 3.3. Tensile strength test

**Figure 3:**
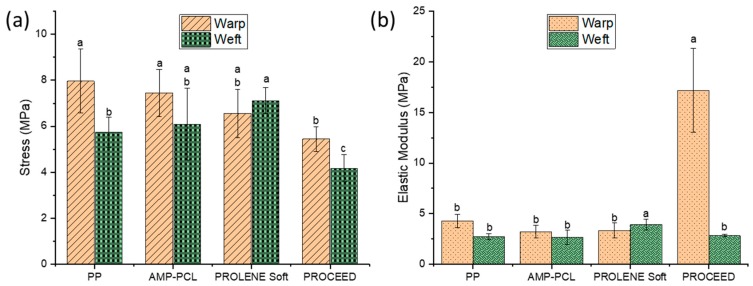
Tensile strength test of samples. (**a**) The max tensile stress; (**b**) the elastic modulus in warp and weft directions. Different letters in panels (a, b, c) indicate statistically significant differences (*p* < 0.05).

The uniaxial tensile strength is the most investigated mechanical property of hernia repair meshes. In the current study, the max stress of AMP-PCL showed no significant difference with commercially available mesh PROLENE Soft in both warp and weft directions (Figure 3a). What is more, the max stress of the warp and weft direction of fabricated mesh, PP and AMP-PCL, are markedly higher than that of the composite mesh PROCEED (*p* < 0.05). Besides, the addition of AMP-containing PCL nanofiber film did not notably affect the max tensile stress of the scaffold in both directions. The elastic modulus of prepared scaffolds and naked PP meshes were at the similar values as the two commercial surgical meshes tested here (Figure 3b). Herein, AMP-PCL composite mesh is appropriate for the proposed application with respect to tensile strength.

### 3.4. In Vitro Release Activity

**Figure 4:**
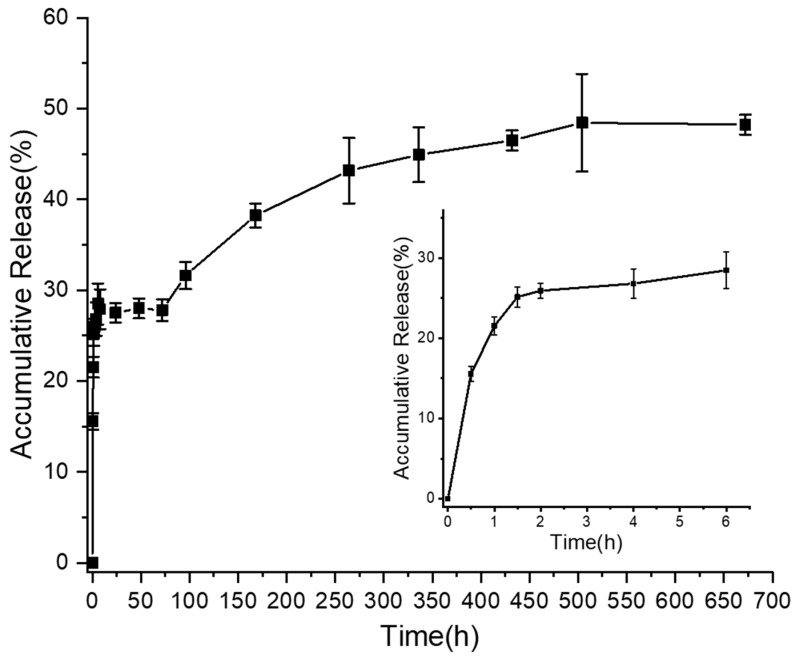
In vitro release profiles of PEP-1 from scaffold in PBS buffer. The inset shows the detailed release behaviors of mesh in the first 6 h. Each data plot was measured from three samples.

The in vitro release properties of composite mesh are illustrated in Figure 4. About 16% of the peptides were released in the first 30 min and over 25% peptides were detected in the PBS buffer after 1.5 h of incubation. The accumulated release amount did not increase from 6 h to 72 h (around 27.5%). After then, peptides seemed to release slowly. Roughly only 48% of the peptides were detected after 28 days of incubation, and the curve tends to be parallel with the horizontal line, suggesting that few peptides were released after 21 days.

### 3.5. In Vitro Antibacterial Activity

**Figure 5:**
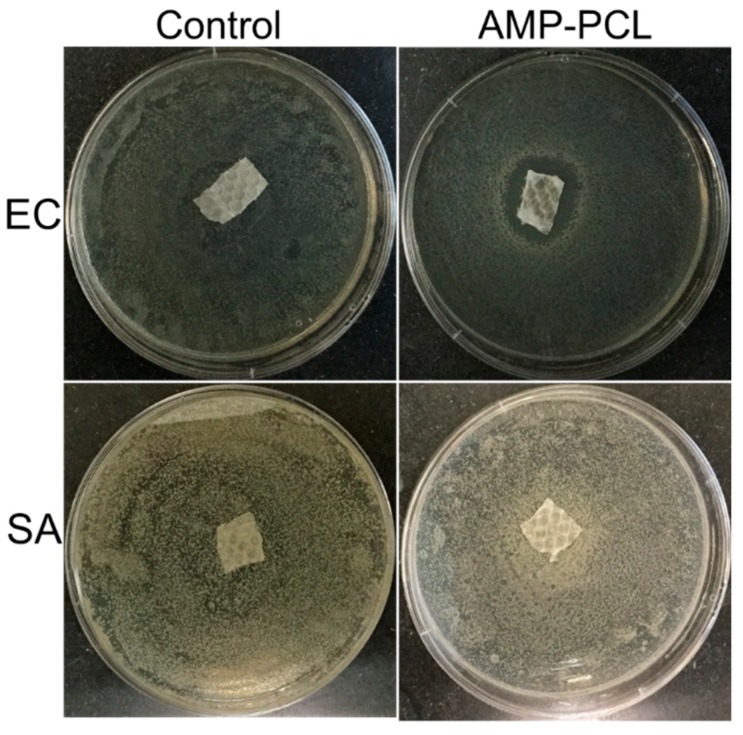
The antibacterial test results against *E. coli* and *S. aureus*.

Infection in hernia repair surgery normally causes severe outcomes; therefore, it is vital to endow antibacterial performance to surgical meshes. The antimicrobial test results of AMP-PCL are shown in Figure 5. Obviously, there was a small inhibition zone (4.76 mm) around AMP-PCL scaffolds in the EC test. However, AMP-PCL showed no inhibition activity against SA. On the contrary, it seems that the proliferation of staphylococci near the modified mesh was improved. The PEP-1 used here was effective against *E. coli*, *S. aureus*, *P. aeruginosa* and *C. albicans* at certain concentrations. The peptide in PCL showed efficacy against EC but none to SA, there are multiple reasons for this that we will discuss later.

### 3.6. In Vitro Cytotoxicity Test

**Figure 6:**
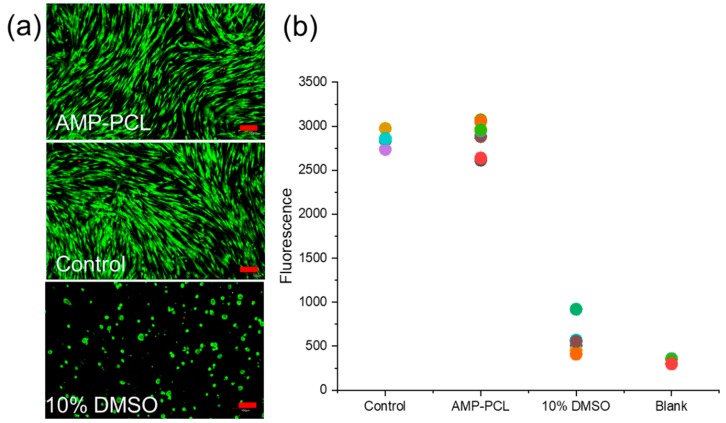
In vitro cytotoxicity evaluation. (**a**) Fluorescent microscopic observation of HDFs cultured in each group. Scale bars = 100 µm. (**b**) Cell proliferation by Alamar blue assay.

As shown in Figure 6a, cells cultured in the leaching liquid were alive and appeared to have the same spread morphology as the control. In the Alamar blue assay, the fluorescence value of AMP-PCL showed no difference to the control (*p* = 0.988) and was significantly higher than that of the cells cultured in 10% DMSO (*p* = 0.000). Therefore, these scaffolds showed no toxicity to human dermal fibroblasts.

## 4. Discussion

Infection at the surgical site during the hernia repair process usually results in surgical failure and brings heavy pain and high costs to patients. Many researches have focused on the fabrication of composite meshes with antibacterial activities in recent years. In the current study, we functionalized mesh with a cationic antimicrobial peptide which we synthesized in our lab and hypothesized that peptides would diffuse from nanofibers and the composite mesh would exhibit antibacterial performances. PEP-1 (RRRGRRRGPPGRRRGRRR) in this study is a cationic hydrophilic short peptide, with low toxicity to mammalian cells and broad-spectrum bactericidal activities.

As shown in the FTIR spectra of all samples (Figure 2), the peaks at 3186 and 3275 cm^−1^ of AMP-PCL are too small compared to the peaks at 1535 and 1632 cm^−1^. We think there are several reasons for this. Firstly, on the PEP-1 spectrum, the peaks at 3186 and 3275 cm^−1^ are smaller than the peaks at 1535 and 1632 cm^−1^. Secondly, the concentration of peptides in AMP-PCL is relatively low. Therefore, the corresponding peaks of Amide I, Amide II and the NH stretching vibration are small.

From the in vitro release activity in Figure 4, we knew that peptides would diffuse from nanofibers and they had a burst release in the first 2 h. What is more, AMP-PCL exhibited inhibition against EC after 48 h incubation, which furtherly confirmed that peptides could leach from nanofibers. However, AMP-PCL did not show inhibition activity to SA. To find out its cause, the peptide loading amount may not be enough, considering that the PEP-1 here is less effective to SA (MBC at 2273 µg/mL) than to EC (MBC at 1136 µg/mL) (see the minimal bactericidal concentration (MBC) in Table S1).

**Figure 7:**
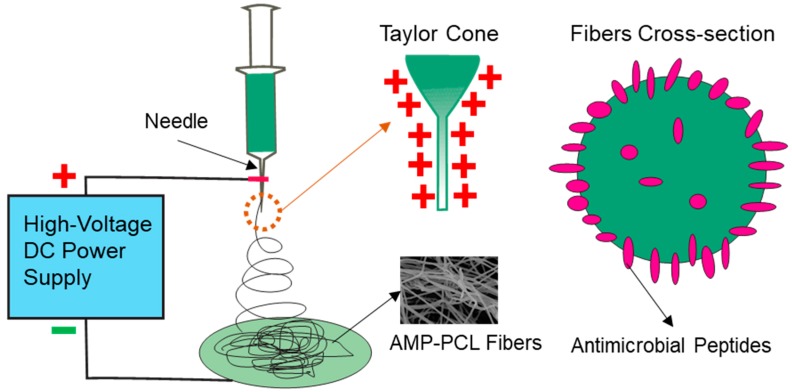
Schematic illustration of antimicrobial peptides distribution during the electrospinning process.

In a previous study [41], nisin was dissolved in N, N-dimethylformamide, mixed with PEO-PDLLA (50:50), and electrospun to form nisin-containing nanofibers. The antimicrobial activity tests showed that these nanofibers inhibited *S. aureus* effectively, and the antibacterial activity lasted for 9 days. However, unlike their study, peptide electrospun in PCL in this study did not behave the antimicrobial activities as it did solely effectively. The reason for this lies in the structural difference between nisin and PEP-1 in this study. PEP-1 here is cationic in net charge (+ 12), more hydrophilic and much shorter in length than nisin (34 amino acid residues). During the electrospinning process, the anode was applied to the syringe needle, and peptide segments would appear at the periphery of nanofibers due to its cationic net charge. Figure 7 illustrates the schematic of this process. This phenomenon was in accordance with a report in 2007, which suggested that positively charged amphiphiles, octadecyl rhodamine B or Rhodamine B, would migrate to the surface of PCL fibers due to the field-driven partition effect [48].

On the other hand, AMP-PCL cannot be sterilized through ultraviolet light or autoclave since the peptides would be inactivated in these conditions. The 75% ethanol in water used to sterilize mesh in this study also caused peptide loss before the antibacterial test. The interactions between peptides and PCL molecules are mainly Van der Waals forces, and PEP-1 is very easy to dissolve in water due to its hydrophilic character. Hence, the peptides located at the periphery of nanofibers are easily diffused from fibers and dissolved in water. We can figure out that only about 10% of the peptides were released from 0.5 h to 48 h from Figure 4, suggesting that most peptides in the surface were released in the first half an hour, and peptides in the internal zone of fibers displayed a relatively slow release rate. Therefore, a large quantity of peptides might be dissolved in the 75% ethanol solution during the sterilization process. As a result, far from enough peptides were effectively leached to inhibit bacteria during antibacterial tests.

From the in vitro antibacterial results in Figure 5, we found that the staphylococci showed an increased growth near the AMP-PCL. This may have resulted from the sub-inhibitory effect. The effect of a sub-inhibitory concentration of a potential biocide (like PEP-1 in this work) on bacteria is complex. It may induce SOS reaction, lead to gene mutation, or reduce the bacteria virulence factor expression [49,50], which is an open issue that needs to be specifically investigated in further studies.

In conclusion, the modified mesh reported in this study exhibited sufficient mechanical properties, inhibition activity against *E. coli*, and no cytotoxicity to human dermal fibroblasts. However, mesh did not inhibit SA in the in vitro antibacterial assay. We found that many AMPs reported were highly toxic to human cells, along with strong antibacterial efficacy. Although the antimicrobial peptide PEP-1 used here was not as highly effective as some peptides reported previously, the PEP-1 was selected mainly due to its low toxicity to mammalian cells. However, the disinfection method should also be considered carefully according to the specific characters of materials. Liquid sterilization conditions are probably not appropriate to disinfect cationic and hydrophilic peptides involved electrospinning fibers similar to this study due to the risk of peptide loss. While under dry sterilization conditions, peptides would exhibit initial burst release from fibers and then a long and slow release phenomenon. On the contrary, neutral antimicrobial agents like nisin may be more suitable to release slowly through this method. Therefore, the properties of antimicrobial agents, the methods of disinfection, and the sample preparation method should all be considered based on the actual situations. Investigations into grafting antimicrobial peptides onto polymers through covalent bonds could be further carried out.

## 5. Conclusions

This study functionalized composite mesh with antibacterial performances by electrospinning antimicrobial peptide with PCL fibers. This mesh proved to be effective against *E. coli* after 48 h incubation and did not show toxicity to human dermal fibroblasts. The mechanical properties of scaffold are also appropriate compared to that of some commercial surgical meshes. Although peptides exhibited an initial burst release in PBS buffer, this method to load antimicrobial peptides to meshes is simple and practicable. Besides, peptides with more functions or that are more effective against bacteria could be studied in the further research.

## Supplementary Materials

The following are available online at https://www.mdpi.com/1996-1944/12/10/1676/s1, Table S1: Antimicrobial activities (MIC and MBC) of the synthesized peptides.
