# Supplementary material for: Preparation and in Vitro Evaluation of New Composite Mesh Functionalized with Cationic Antimicrobial Peptide
Source: Materials (Basel). 2019 May 23;12(10):1676. doi: 10.3390/ma12101676 (PMC6566986; doi:10.3390/ma12101676)
Supplement: Supplementary file 1 [file materials-12-01676-s001.pdf]

materials

Supplementary Materials

Preparation and in Vitro Evaluation of New Composite Mesh Functionalized with Cationic Antimicrobial Peptide

Pengbi Liu [1,2], Nanliang Chen [1,*], Jinhua Jiang [1,*] and Xuejun Wen [2,3,4,*]

[1] College of Textiles, Donghua University, Shanghai 201620, P. R. China; 1135009@mail.dhu.edu.cn
[2] Department of Chemical and Life Science Engineering, School of Engineering, Virginia Commonwealth University, Richmond, VA 23284, USA; xwen@vcu.edu
[3] School of Pharmaceutical Sciences, Guangzhou Medical University, Guangzhou, 511436, P. R. China
[4] Beijing Ditan Hospital, Capital Medical University, Beijing, 100015, P. R. China
* Correspondence:nlch@dhu.edu.cn (N.C.); jiangjinhua@dhu.edu.cn (J.J.)

Received: 26 April 2019; Accepted: 20 May 2019; Published: date

1. Peptide synthesis

Peptide PEP-1 was synthesized on a Focus Xi Peptide Synthesizer (AAPPTec, LLC, USA) using standard Fmoc-solid phase peptide synthesis protocol. Briefly, 15 mL DMF was added into the reaction vessel (RV) with resin (1 g, 0.57 mmol) and stayed for 40 min. Then 15 mL 20 % piperidine in DMF was added into the RV to remove the N-α protecting group for 40 min. After washed the resin thoroughly with DMF, a solution of the first amino acid Fmoc-Arg (pbf)-OH (1.11 g, 1.71 mmol), HBTU (4.3 mL, 1.71 mmol), DIEA (1.71 mL, 3.42 mmol) and DMF (9 mL) were added to the RV and agitated for 70 min. The first amino acid was grafted to the resin. Then the deprotected process and coupling reactions were continued on until the last amino acid was attached. After the resin was dried, 15 mL cleavage solution were used to obtain crude peptides. The cleavage solution we used consisted of 82.5% TFA, 1% TIS, 5% DI water (Milli-Q A10, Millipore, USA), 1.5% EDT, 5% thioanisole and 5% phenol.

2. RP-HPLC analysis and purification

Crude peptide was analyzed utilizing a C18 analytical column (5 μm, AAPPTec, LLC, USA). Two mobile phases were used. Phase A contained 99.9 % DI water and 0.1% TFA, and phase B contained 99.9% acetonitrile and 0.1% TFA. For the unknown peptides, the elution strength increased by changing phase B from 2 to 80% over 20 minutes at a flow rate of 1.0 mL/min and detection at 214 nm. Then the elution strength was adjusted by changing the starting and ending composition of the two mobile phases and the elution time until the prominent peaks were separated from other poor peaks. The main peak was separated and collected on the preparative HPLC column (C18, 10 μm, AAPPTec, LLC, USA) using the similar elution gradient but detection at 220 nm. Lyophilized the main peaks and stored the purified peptides in a freezer.

3. Antimicrobial activities

The antimicrobial activities Minimum Inhibitory Concentrations (MIC) and Minimum Bactericidal Concentration (MBC) of PEP-1 were determined with slight modification of standard methods [1,2]. PEP-1 was diluted into a serial of concentrations, namely 10mg/ml, 5000μg/ml, 2500 μg/ml, 1250 μg/ml, 625 μg/ml, 313 μg/ml, 156 μg/ml, 78 μg/ml, 39 μg/ml, and 20μg/ml. Bacteria were cultured in these solutions for 24 h at 37 °C and the MIC was the lowest concentration that looked clear with naked eyes. While the clear groups were selected to test MBC. Clear solutions were spread onto agar plates and incubated for 24 h at 37 °C. The number of colony forming units (CFU) on each plate was counted. The MBC is identified by determining the lowest concentration of peptides that

reduces the viability of the initial bacterial inoculum by a pre-determined reduction of ≥ 99.9 % (n= 3).

Table S1. Antimicrobial activities (MIC and MBC) of the synthesized peptides.

Peptide	MIC (μg/ml)				MBC (μg/ml)			
	P. aeruginosa	***E. coli***	***S. aureus***	***C. albicans***	***P. aeruginosa***	***E. coli***	***S. aureus***	***C. albicans***
PEP-1	1,136	285	285	1,136	1,136	1,136	2,273	2,273

References

1. Ilker, M.F.; Nüsslein, K.; Tew, G.N.; Coughlin, E.B. Tuning the hemolytic and antibacterial activities of amphiphilic polynorbornene derivatives. *J. Am. Chem. Soc.* **2004**, *126*, 15870–15875.
2. King, A.; Chakrabarty, S.; Zhang, W.; Zeng, X.; Ohman, D.E.; Wood, L.F.; Abraham, S.; Rao, R.; Kenneth J. Wynne, K.J. High antimicrobial effectiveness with low hemolytic and cytotoxic activity for PEG/quaternary copolyoxetanes. *Biomacromolecules* **2014**, *15*, 456–467.

© 2019 by the author. Licensee MDPI, Basel, Switzerland. This article is an open access article distributed under the terms and conditions of the Creative Commons Attribution (CC BY) license (http://creativecommons.org/licenses/by/4.0/).
